# Association between Influenza Vaccination and Positive SARS-CoV-2 IgG and IgM Tests in the General Population of Katowice Region, Poland

**DOI:** 10.3390/vaccines9050415

**Published:** 2021-04-21

**Authors:** Małgorzata Kowalska, Ewa Niewiadomska, Kamil Barański, Angelina Kaleta-Pilarska, Grzegorz Brożek, Jan Eugeniusz Zejda

**Affiliations:** 1 Department of Epidemiology, School of Medical Sciences in Katowice, Medical University of Silesia in Katowice, 40-752 Katowice, Poland; mkowalska@sum.edu.pl (M.K.); akaleta@sum.edu.pl (A.K.-P.); gbrozek@sum.edu.pl (G.B.); jzejda@sum.edu.pl (J.E.Z.); 2 Department of Epidemiology and Biostatistics, School of Public Health in Bytom, Medical University of Silesia in Katowice, 41-902 Katowice, Poland; eniewiadomska@sum.edu.pl

**Keywords:** influenza vaccination, seropositivity of COVID-19, cross-sectional study

## Abstract

The explanation of the potential interaction between the influenza vaccine and SARS-CoV-2 infection is urgently needed in the public health. The objective of the study is to compare the occurrence of positive SARS-CoV-2 IgG and IgM tests in subjects with and without recent (last year) seasonal influenza vaccinations. In a cross-sectional study located in three large towns of Silesian Voivodeship (Poland), we studied 5479 subjects in which 1253 (22.9%) had a positive anti-SARS-CoV-2 IgG test and 400 (7.3%) had a positive anti-SARS-CoV-2 IgM test. Seasonal influenza vaccination remains an independent factor protecting against positive IgG tests (OR = 0.68; 0.55–0.83). The effect is not apparent with IgM antibodies. The obtained results confirmed that the serological status of SARS-CoV-2 infection depends on vaccination against seasonal influenza.

## 1. Introduction

Influenza and COVID-19 are respiratory viral illnesses that may present with similar symptoms, and coinfections can result in more serious complications with fatal outcomes [1]. Both viruses, (i.e., influenza and the novel coronavirus) depend on a viral RNA polymerase and use surface proteins to infect the host [2]. The potential interaction between the influenza vaccine and SARS-CoV-2 infection has attracted the attention of some researchers. Moreover, authors from the Netherlands suggest that a quadrivalent inactivated influenza vaccine can induce trained humoral immunity in an organism or other mechanisms through which an enhanced antiviral state is acquired after vaccination [3]. Some current published data suggest that flu vaccinations can augment immunity against other viral infections, such as SARS-CoV-2 [4]. Humoral immune responses to SARS-CoV-2 are mediated by antibodies that are directed to viral surface proteins, mainly the spike glycoprotein and the nucleocapsid protein, and such antibodies neutralize the viral infection of human cells [5]. Annual influenza vaccination is recommended to protect against infection with seasonal influenza viruses [6]. Discussion about the potential benefits or risks of influenza vaccination on the risk of COVID-19 persists, although there is no clear scientific explanation for a possible effect of influenza vaccination on the risk of SARS-CoV-2 infection [7,8,9,10,11]. The results of a cohort study among Spanish healthcare workers suggested that influenza vaccinations do not significantly modify the risk of SARS-CoV-2 infection. The adjusted odds ratio was OR = 1.07; 95% CI, 0.92–1.24 and OR = 0.86; 95% CI, 0.68–1.08, respectively, in the whole group and in symptomatic patients [12]. However, in the EPICOVID19 questionnaire study conducted in Italy, influenza vaccinations were associated with a decreased probability of a SARS-CoV-2-positive test in the younger participants (OR = 0.85, 95% CI, 0.74–0.98) [13]. Other authors have posited the minimum number of influenza vaccinations needed to obtain herd immunity [14].

The objective of the study is to compare the occurrence of positive SARS-CoV-2 IgG and IgM tests in subjects with and without recent (last year) seasonal influenza vaccination. The hypothesis assumes that the frequency of positive IgG and IgM tests is lower in people vaccinated against seasonal influenza than in nonvaccinated.

## 2. Materials and Methods

The study was performed as a cross-sectional seroepidemiological survey. It was located in three Silesian Voivodeship towns: Katowice, Gliwice, and Sosnowiec, with an estimated summary population of 694,000 inhabitants, which represents about 33% of the source populations. The study protocol was described at the ClinicalTrials.gov PRS system website, and the project was given a registration number: ClinicalTrials.gov Identifier: NCT04627623 [15]. Having obtained names and postal addresses, we sent invitations to 6000 people. The sample size calculation was performed for obtaining the required number of participants (Supplementary S1). The first invitation letter was sent in August and September, and in October, all selected participants received a second invitation as a reminder. Only 1167 people (19.5% of those invited) expressed written consent to participate in the study. Ultimately, we introduced a supplementary recruitment in which we obtained 4312 spontaneous applicated residents of the selected cities (74.1% of the 5815 people willing to be tested for antibodies). The final group included 5479 residents (91.3% of the assumed number of people); age and sex distribution of the study group did not differ from the distribution in the general population of the Silesian Voivodeship [16] (Supplementary Figure S1).

All participants underwent questionnaires and laboratory examinations in local laboratories affiliated within the network “Diagnostyka”. The questionnaire included demographical questions, as well as questions on COVID-19 diagnosis, symptoms suggestive of a viral infection in the period preceding IgG and IgM antibodies measurement, and questions on the history of flu vaccination in the last year (yes/no/I do not know). The questionnaire was adopted and based on WHO protocol [17] and presented in Supplementary S2. At each local laboratory, blood samples were tested using a semiquantitative commercial test kit ELISA (EuroImmun Polska Sp z o.o., Wrocław, Poland). Antibodies IgG and IgM were measured against the S1 proteins of SARS-CoV-2 in serum, and the results were expressed as ratios (test/control extinction), according to the following scale: ratio <0.8 = negative result, ratio 0.8–1.09 = questionable result, and ratio >1.09 = positive result (the specificity and maximum sensitivity of the IgG test was 99% and 88%, respectively). Ethical approval for this study was obtained from Ethics Committee of Medical University of Silesia in Katowice (no PCN/0022/KB1/61/20), with the date of approval 14/07/2020.

Statistical analyses were performed using the procedures of the R statistical package v.3.6.2 (2019, The R Foundation for Statistical Computing, GNU General Public License; The Comprehensive R Archive Network). The distribution of quantitative variables was presented as the mean values and their standard deviations, and the categorical values (such as the prevalence of positive IgG and IgM tests) were presented as absolute (n) and relative frequencies (%). Between-group differences in the distribution of qualitative variables were tested by the chi-square (or Fisher’s) test. Moreover, to assess the relationship between the positive anti-SARS-CoV-2 result and declared seasonal influenza vaccination, the crude odds ratio and 95% CI (confidence interval) were used. Adjusted odds ratios were verified using multiple logistic regression in which IgM or IgG results, such as the dependent variable and sex, age, declared diseases, obesity and/or overweight, previous COVID-19 contact or quarantine, and influenza vaccination, were the explanatory variables. We checked the collinearity of the independent variables used in the model, and we did not identify the autocorrelation between them. Finally, a backward stepwise regression was used to assess the relationship between the positive results of the antibodies test and the particular classification variables. In the interpretation of the results, *p*-values below 0.05 were considered statistically significant.

## 3. Results

In the group of 5479 subjects, 1253 (22.9%) had a positive anti-SARS-CoV-2 IgG test, and 400 (7.3%) had a positive anti-SARS-CoV-2 IgM test. Vaccinations against influenza in the last year were reported in 903 (16.5%) of the study participants. Table 1 shows sex, age, COVID-19-related history, and occurrence of chronic diseases according to the influenza vaccination status.

The distribution of sex was similar in both groups (*p* = 0.2). The groups that were compared also showed similarities in terms of the frequency of obesity/overweight (*p* = 0.4), COVID-19-related history (*p* = 0.3), the occurrence of diabetes (*p* = 0.06), and autoimmune diseases (*p* = 0.6). Influenza vaccinated subjects were statistically significantly older (*p* < 0.0001) and more frequently declared the occurrence of hypertension (*p* < 0.0001), chronic allergies (*p* = 0.03), and comorbidities (*p* = 0.02).

The compared groups differed in terms of the seropositivity status (Table 2). The positive SARS-CoV-2 IgG test was statistically significant (*p* < 0.0001) more frequently in nonvaccinated than in vaccinated subjects (22.0% and 15.6%, respectively). The occurrence of positive SARS-CoV-2 IgM tests was similar in both groups (vaccinated subjects: 4.5% and nonvaccinated subjects: 5.1%; *p* = 0.7). In the subgroups defined by sex or age, there was a similar association between the influenza vaccination status and positive SARS-CoV-2 antibody tests (Table 2). Moreover, the frequency of questionable results (Ques) of the IgG antibodies test concerned 103 subjects (1.9%) and, in the case of IgM antibodies, respectively, 125 subjects (2.3%). In a further analysis connected with the categories “questionable” and “negative”, the results of the test were grouped into two categories: positive results or nonpositive results (Table 3).

Table 3 shows the estimated crude odds ratios, revealing that the seasonal influenza vaccination in the year preceding the study had a protective effect on the occurrence of positive IgG test results in the total group, as well as in the subgroups defined by sex, age, a history of chronic diseases, and previous COVID-19 contact or quarantine.

**Figure 1:**
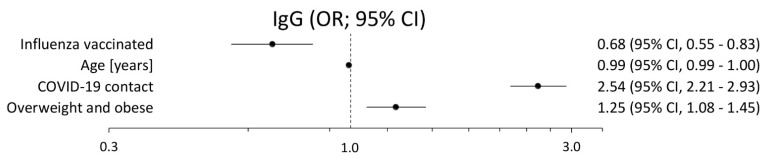
Results of the multivariable analysis (adjusted odds ratio and its 95% confidence interval) for the relationship between the positive results of the IgG test and particular classification variables.

**Figure 2:**
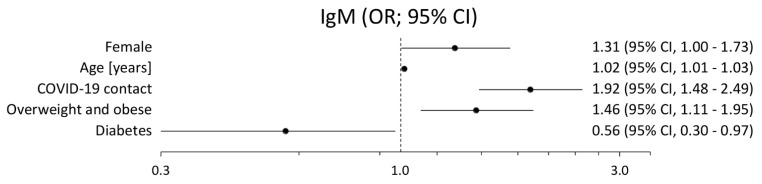
Results of the multivariable analysis (adjusted odds ratio and its 95% confidence interval) for the relationship between the positive results of the IgM test and particular classification variables.

Figure 1 and Figure 2 present the results of the backward stepwise regression used to assess the relationship between the positive results of the IgG or IgM test and particular classification variables. In the case of IgG antibodies, the risk of a positive test was more than twice as high among subjects with declared previous COVID-19 contact or quarantine (OR = 2.54; 2.21–2.93). Statistically significant relationships were also confirmed in obese or overweight people (OR = 1.25; 1.08–1.45). On the other hand, seasonal influenza vaccinations remain an independent factor protecting against positive IgG tests (OR = 0.68; 0.55–0.83). Interestingly, older age is protective of the assessed relationship (OR = 0.993; 0.988–0.997). In the case of IgM, a positive antibodies test occurred significantly often in females (OR = 1.31; 1.003–1.732), in older age (OR = 1.02; 1.01–1.03), in overweight or obese people (OR = 1.46; 1.11–1.95), and in people with previous COVID-19 contact or quarantine (OR = 1.92, 1.48–2.49).

## 4. Discussion

The SARS-CoV-2 virus and influenza virus share transmission routes, infections have common clinical manifestations, and both infections may coexist. A Chinese study performed at the beginning of the pandemic showed that 57% out of 307 SARS-CoV-2 infected patients were positive for influenza viruses [18]. However, several other studies did not confirm frequent coinfections [19,20].

A similar etiopathogenic background of both diseases provokes important practical questions concerning coexisting influenza and SARS-CoV-2 infections. From an epidemiological point of view, the answers to such questions are important because of the overlap of both epidemics in a real-life setting [21]. One question addresses the impact of coexisting influenza on the severity of SARS-CoV-2 infection. A large study performed in England showed that coinfections increase the risk of COVID-19 death by 2.4 [22].

Another question is whether existing influenza infections increase the risk of SARS-CoV-2 infection. Although there is no convincing evidence from human studies, the findings of recently published cellular and animal experiments show that infection with the influenza virus enhances SARS-CoV-2 infectivity [23]. The authors suggest that influenza vaccination should be recommended to people with a high risk of SARS-CoV-2 infection. The results of our study showed in a population setting that the occurrence of anti-SARS-CoV-2 IgG antibodies was statistically significantly less frequent in people who had undergone vaccination for seasonal influenza (15.6% vs. 22.0%). No similar effect was found in IgM antibodies. The protective effect of vaccination (OR = 0.68; 95% CI 0.55–0.83) was seen after the adjustment for age, sex, history of COVID-19 contacts, and chronic health conditions.

Our findings are in line with the results of the ecological study in Italy that showed an inverse association between influenza vaccination coverage and SARS-CoV-2 seroprevalence [7]. Other studies have reported the same directionality of the relationship between influenza vaccination prevalence and COVID-19 hospitalizations and mortality [24]. The ecological study in the USA reported a protective effect of the influenza vaccine on COVID-19 mortality in the elderly [9]. The results showed that each 10% increase in vaccination coverage was associated with a 28% decrease in the COVID-19 death rate. Another American study showed that SARS-CoV-2-positive patients appear to be less symptomatic and have a less severe course of COVID-19 if they already received a seasonal influenza vaccine in the current flu season and were vaccinated for pneumococcal vaccines [25]. However, in the authors’ opinion, such an effect could result from the more restrictive lifestyle of vaccinated people during the COVID-19 pandemic.

An important association of SARS-CoV-2 seropositivity and vaccination against influenza was observed in a large study (over 27,000 patients) performed in Michigan (USA). Compared with nonvaccinated patients, the vaccinated group showed a less frequent occurrence of positive tests (OR = 0.82; 95% CI 0.73–0.92), a lower rate of hospitalizations, and a shorter length of in-hospital stay, as well as a decreased need for mechanical ventilation [26].

There are several potential explanations for the protective effect of influenza vaccination against COVID-19. Firstly, it could reflect a selection mechanism. People who observe health-driven precautions might be more likely to accept influenza vaccinations, and at the same time, they might be more likely to effectively follow public health recommendations during the pandemic. Other mechanisms might be related to vaccination-induced changes in the nonspecific immune response. Moreover, the results of the recent studies showed that people vaccinated against influenza have a higher willingness to get vaccinated against SARS-CoV-2, which is a very positive phenomenon in public health [27,28].

The T cell-mediated response is believed to play an important role. It cannot be excluded that vaccination against influenza induces a weaker, virus-specific CD8 T-cell immune response compared to the case of natural infection. As a result, T cells develop more diversity, which increases the potential for protection against other infections. Unvaccinated individuals are more likely to have a higher proportion of influenza-specific resident memory T cells in the lungs, which are highly productive of inflammatory cytokines. This might be associated with the exaggerated inflammatory response and severe Acute Respiratory Distress Syndrome observed in some COVID-19 patients [9]. The protocols used in our study do not allow for the interpretation of the underlying mechanisms of the protective effect of influenza vaccination against SARS-CoV-2 infection.

Other statistically significant correlations of IgG seropositivity found in our study were age, contact with COVID-19 patients, and overweight/obesity. For IgM seropositivity, the additional statistically significant correlation was diabetes. The effect of age in association with the influenza vaccination is also known from mortality studies. The study in Brazil showed that, in nonvaccinated patients, the COVID-19 mortality was 14% in children and 84% in the elderly, whereas, in vaccinated patients, the COVID-19 mortality was much lower [29]. Moreover, obesity and diabetes were statistically significant risk factors for COVID-19-related mortality in this study. From an epidemiological point of view, it is important that the immune response to SARS-CoV-2 infection is heterogeneous and varies between individuals based on age, environment, and underlying health conditions [16,29,30,31]. The individual profile of the immune response is pertinent in shaping the risk of infection. Unlike studies reporting on the association between influenza vaccination and COVID-19 morbidity and mortality, our study focused on the serological manifestation of SARS-CoV-2 infection in the population setting. We assessed the coronavirus infection status using the measurement of anti-SARS-CoV-2 IgG seropositivity. Moreover, the study was performed using individual data obtained via a cross-sectional design. However, there were some pertinent study limitations. First, the data on the history of vaccination against influenza were collected using a questionnaire, and their true reliability remains unknown, as in other questionnaire-based epidemiological studies. Moreover, we did not collect information on the exact date or type of influenza vaccination, although seasonal influenza vaccinations offered in the second half of 2020 were inactivated quadrivalent vaccines. [11]. Due to these limitations, we recommend further studies needed to confirm a detailed association between the type and date of influenza vaccination and positive results of anti-SARS-CoV-2. Furthermore, recruitment is a possible limitation of this study. For example, the first group was randomly selected, and the second included a supplementary sample in which there was more female participants, as well as people who declared previous contact with COVID-19 and seasonal influenza-vaccinated people (12.2% vs. 17.6%, respectively), as well as people without declared chronic diseases. Both groups had similar profiles concerning age. We plan to publish those observations soon in a methodological article.

## 5. Conclusions

In conclusion, our findings suggest that a reported history of vaccination against seasonal influenza was negatively associated with SARS-CoV-2 infection, as evidenced by IgG, but not IgM, antibodies in the spike protein (OR = 0.68; 0.55–0.83).

## Figures and Tables

**Table 1:** Sex, age, COVID-19 related history, and the occurrence of chronic diseases, according to the influenza vaccination status (relative values in the parentheses).

Influenza Vaccination in Last Year		Total 5479	No 4576 (100) *	Yes 903 (100) *	*p*-Value
Sex	Male	2287	1895 (41.4)	392 (43.4)	0.2 ^1^
	Female	3192	2681 (58.6)	511 (56.6)	
Age (years)		43.9 ± 16.8	43.1 ± 16.6	47.7 ± 17.6	*p* < 0.0001 ^2^
Body Mass Index Overweight and obese	No	2382	2001 (47.4)	381 (46.0)	0.4 ^1^
	Yes	2669	2221 (52.6)	448 (54.0)	
Contact with COVID-19 or quarantine	No	3618	3035 (66.3)	583 (64.6)	0.3 ^1^
	Declared	1861	1541 33.7)	320 (35.4)	
Hypertension	No	4138	3510 (76.7)	628 (69.5)	*p* < 0.0001 ^1^
	Declared	1341	1066 (23.3)	275 (30.5)	
Diabetes	No	5126	4294 (93.8)	832 (92.1)	0.06 ^1^
	Declared	353	282 (6.2)	71 (7.9)	
Chronic allergy	No	4851	4071 (89.0)	780 (86.4)	0.03 ^1^
	Declared	628	505 (11.0)	123 (13.6)	
Autoimmune diseases	No	5112	4266 (93.2)	846 (93.7)	0.6 ^1^
	Declared	367	310 (6.8)	57 (6.3)	
Comorbidity (two or more coexisting diseases)	No	4714	3960 (86.5)	754 (83.5)	0.02 ^1^
	Declared	765	616 (13.5)	149 (16.5)	

Legend: ^1^—*p*-results of the χ2 test for the categorical values, ^2^—*p*-results of the Mann–Whitney *U* test for quantitative values, and *—analysis performed excluding the missing values.

**Table 2:** Association between the SARS-CoV-2 serological status and influenza vaccinations in all subjects and their specific subgroups defined by sex and age and COVID-19-related history.

Influenza Vaccination in Last Year	Total Study Group (N = 5479)							
	Anti-SARS-CoV-2 Antibodies IgG *N* (%)				Anti-SARS-CoV-2 Antibodies IgM *N* (%)			
	Neg	Ques	Pos	*p* Value	Neg	Ques	Pos	*p* Value
Yes 903 (100)	753 (83.4)	9 (1)	141 (15.6)	<0.0001	842 (93.2)	20 (2.2)	41 (4.5)	0.7
No or I do not know 4576 (100)	3473 (75.9)	94 (2.1)	1009 (22.0)		4237 (92.6)	105 (2.3)	234 (5.1)	
	**Male subjects (*N* = 2287)**							
Yes 392 (100)	328 (83.7)	1 (0.3)	63 (16.0)	0.0007	369 (94.1)	5 (1.3)	18 (4.6)	0.6
No or I do not know 1895 (100)	1449 (76.4)	39 (2.1)	407 (21.5)		1774 (93.6)	38 (2.0)	83 (4.4)	
	**Female subjects (*N* = 3192)**							
Yes 511 (100)	425 (83.2)	8 (1.6)	78 (15.2)	0.0008	473 (92.5)	15 (2.9)	23 (4.5)	0.5
No or I do not know 2681 (100)	2024 (75.5)	55 (2.0)	602 (22.5)		2463 (91.9)	67 (2.5)	151 (5.6)	
	**Subjects aged below 65 years (*N* = 4741)**							
Yes 721 (100)	588 (81.6)	9 (1.2)	124 (17.2)	0.0006	674 (93.5)	15 (2.1)	32 (4.4)	0.9
No or I do not know 4020 (100)	3016 (75)	89 (2.2)	915 (22.8)		3739 (93)	89 (2.2)	192 (4.8)	
	**Subjects aged 65+ years (*N* = 681)**							
Yes 171 (100)	155 (90.6)	0 (0)	16 (9.4)	0.05	159 (93)	5 (2.9)	7 (4.1)	0.2
No or I do not know 510 (100)	425 (83.3)	3 (0.6)	82 (16.1)		458 (89.8)	13 (2.5)	39 (7.7)	
**Subjects with contact with COVID-19 or quarantine (*N* = 3618)**								
Yes (100)	510 (87.5)	4 (0.7)	69 (11.8)	0.004	552 (94.7)	12 (2.1)	19 (3.2)	0.5
No or I do not know (100)	2495 (82.2)	57 (1.9)	483 (15.9)		2862 (94.3)	50 (1.6)	123 (4.1)	
**Subject without contact with COVID-19 or quarantine (*N* = 1861)**								
Yes (100)	243 (75.9)	5 (1.6)	72 (22.5)	*p* < 0.0001	290 (90.6)	8 (2.5)	22 (6.9)	0.6
No or I do not know (100)	978 (63.5)	37 (2.4)	526 (34.1)		1375 (89.2)	55 (3.6)	111 (7.2)	

Legend: Neg—Negative, Pos—Positive, Ques—Questionable, ^1^—*p*-results of the χ2 test, and ^2^—*p*-results of the Fisher’s test.

**Table 3:** Crude odds ratio and its 95% confidence interval (CI) for the relationship between seasonal influenza vaccination (“Yes” vs. “No or I Do Not Know”) and the results of the SARS-CoV-2 IgG and IgM tests.

Classification Variable (Stratum)	Odds Ratio (95%CI)	
	IgG Antibodies (Positive vs. Nonpositive ^1^)	IgM Antibodies (Positive vs. Nonpositive ^1^)
Total population	0.65 (0.54–0.79)	0.88 (0.62–1.23)
Male	0.70 (0.52–0.93)	1.05 (0.60–1.73)
Female	0.62 (0.48–0.80)	0.79 (0.49–1.21)
Younger (<65 years)	0.70 (0.57–0.86)	0.93 (0.62–1.34)
Older (65+ years)	0.54 (0.29–0.93)	0.51 (0.21–1.11)
Overweight and obese	0.73 (0.56–0.94)	0.74 (0.45–1.16)
Declared previous contact with COVID-19 or quarantine	0.56 (0.42–0.74)	0.95 (0.58–1.50)
Declared hypertension	0.63 (0.43–0.91)	0.66 (0.34–1.16)
Declared diabetes	0.34 (0.12–0.82)	0.29 (0.02–1.52)
Declared chronic allergy	0.47 (0.26–0.82)	0.18 (0.02–1.35)
Declared autoimmune diseases	0.19 (0.04–0.53)	0.27 (0.01–1.36)
Declared comorbidity (two or more coexisting diseases)	0.54 (0.31–0.90)	0.43 (0.13–1.10)

Legend: ^1^—Nonpositive anti-SARS-CoV-2 test (in presented analysis) includes negative and, also, questionable results.

## Data Availability

The data is available on the request in the Department of Epidemiology, Medical University of Silesia in Katowice. The request should be formulated and send to epikat@sum.edu.pl.

## Supplementary Materials

The following are available online at https://www.mdpi.com/article/10.3390/vaccines9050415/s1, Supplementary S1: Sample size calculation; Figure S1: Age structure of Silesian population and compared groups (random and supplementary sample); Supplementary S2: Seroepidemiological questionnaire for COVID 19 infections.
