# Supplementary material for: Association between Influenza Vaccination and Positive SARS-CoV-2 IgG and IgM Tests in the General Population of Katowice Region, Poland
Source: Vaccines (Basel). 2021 Apr 21;9(5):415. doi: 10.3390/vaccines9050415 (PMC8143078; doi:10.3390/vaccines9050415)
Supplement: Supplementary file 1 [file vaccines-09-00415-s001.zip › Supplementary Material.pdf]

vaccines

MDPI

Supplementary matterial

Association between influenza vaccination and positive SARS-CoV-2 IgG and IgM tests in the general population of Katowice Region, Poland

Małgorzata Kowalska [1], Ewa Niewiadomska [2], Kamil Barański [1,*], Angelina Kaleta-Pilarska [1], Grzegorz Brożek [1] and Jan Eugeniusz Zejda [1]

Supplementary S1: Simple size calculation

Within the Silesian voivodeship region, three towns were chosen: Katowice (pop. 301 000), Gliwice (pop. 184 000), and Sosnowiec (pop. 209 000). The total number of inhabitants in these towns represents 33% of the source population. For each town, a separate sample size was estimated assuming the expected 10% frequency of positive IgG tests, a 3% margin of error, and a 95% confidence level. Given the assumptions, the minimum sample size for each town was 384 subjects, resulting in a total minimum sample size of 1152 subjects. Assuming a limited participation, we decided to select an age-stratified sample of 2000 subjects in each town, using the following age structure (age groups in years) obtained from the national statistical reports: 0-6: 6.4%; 7-14: 8.4%; 15-18: 3.7%; 19-25: 7.3%; 26-35: 14.2%; 36-65: 41.1%; 66-75: 10.9%; 76+: 8.0%. This age structure with equal participation of male and female subjects in each age group was used to select study participants from the records at the central statistical office in Poland. Having obtained names and postal addresses we sent invitations and other required forms (informed written consent, forms required under General Data Protection Regulation, list of laboratories in the towns). The invitation letter was sent in August – September and in October all selected persons received a second invitation, as a reminder. Of 6000 invited persons only 1167 persons responded to the invitation (participation rate: 19.5%), 394 in Katowice, 392 in Gliwice, and 381 in Sosnowiec. Age distribution of the study group (n=1167) did not differ statistically significantly from the age distribution of the general population of the Silesian Voivodeship (p=0.8; Figure below). Detailed data we included in the publication titled 'Seroprevalence of anti SARS-CoV-2 antibodies in a random sample of inhabitants of Katowice Region, Poland' [1].

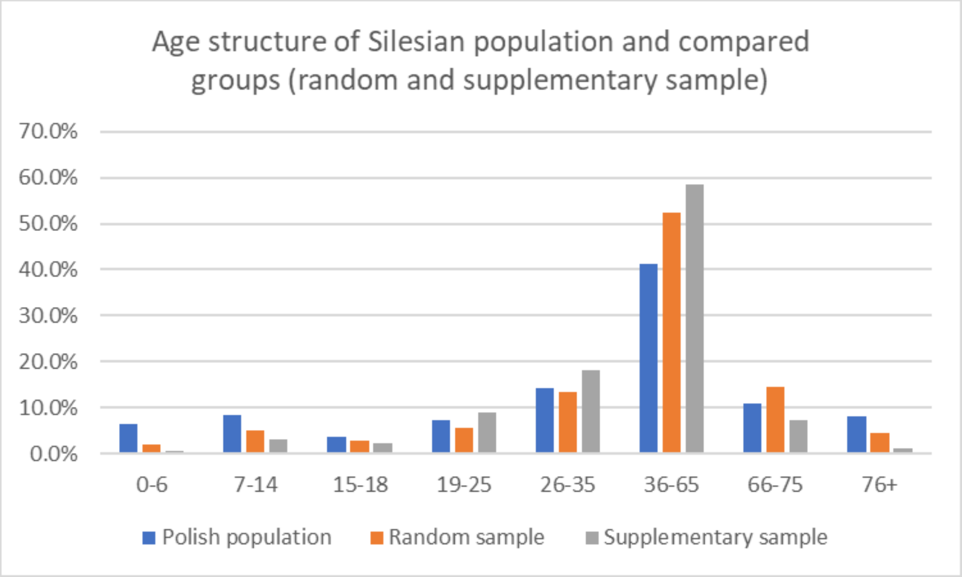

Figure S1. Age structure of Silesian population and compared groups (random and supplementary sample).

Supplementary S2. Seroepidemiological questionnaire for COVID 19 infections (based on WHO recommendation [2].

Report form for all participants

**Unique ID**	
**1. Investigator personal data**	
**Name**	
**Institution**	
**Phone number**	
**Email**	
**Date of interview (DD/MM/YYYY)**	___/____/______
**2. Participant's data**	
**First name**	
**Surname**	
**Sex**	□Male □Female □Not known
**Date of birth (DD/MM/YYYY)**	___/____/______
**Telephone number**	
**Age (years, months)**	
**Email**	
**Country of residence**	
**Nationality**	
**Ethnicity (optional)**	
**Occupation**	
**Have you had contact with anyone with suspected or confirmed COVID-19 virus infection?**	□Yes □No □Unknown<br>If Yes, please, indicate the date __/____/____
**3. Symptoms**	
**In the past (x) months, have you had any of the following symptoms:**<br>**COMMENT: (x) period to cover the time since the emergence of COVID-19 virus to date of data collection**	
**Fever ≥38°C**	□Yes □No □I don’t know
**Chills**	□Yes □No □I don’t know
**Fatigue**	□Yes □No □I don’t know
**Muscle ache (myalgia)**	□Yes □No □I don’t know
**Sore throat**	□Yes □No □I don’t know
**Cough**	□Yes □No □I don’t know
**Runny nose (rhinorrhoea)**	□Yes □No □I don’t know
**Shortness of breath (dyspnoea)**	□Yes □No □I don’t know
**Wheezing**	□Yes □No □I don’t know
**Chest pain**	□Yes □No □I don’t know

**Other respiratory symptoms**	**□Yes**	**□No**	**□I don't know**
**Headache**	**□Yes**	**□No**	**□I don't know**
**Nausea/vomiting**	**□Yes**	**□No**	**□I don't know**
**Abdominal pain**	**□Yes**	**□No**	**□I don't know**
**Diarrhea**	**□Yes**	**□No**	**□I don't know**
**Did any of these symptoms require you to seek medical help?**	**□Yes**	**□No**	**□I don't know**
**Did any of these symptoms require you to be hospitalized?**	**□Yes**	**□No**	**□I don't know**
**Have you ever been vaccinated against tuberculosis?**	**□Yes**	**□No**	**□I don't know**
**Have you been vaccinated last year against influenza?**	**□Yes**	**□No**	**□I don't know**

References

1. Zejda, J.E.; Brożek, G.M.; Kowalska, M.; Barański, K.; Kaleta-Pilarska, A.; Nowakowski, A.; Xia, Y.; Buszman, P. Seroprevalence of Anti-SARS-CoV-2 Antibodies in a Random Sample of Inhabitants of the Katowice Region, Poland. *Int. J. Environ. Res. Public Health* **2021**, *18*, 3188, doi:10.3390/ijerph18063188.
2. WHO. Population-Based Age-Stratified Seroepidemiological Investigation Protocol for COVID-19 Virus Infection. March 2020. Available online: https://apps.who.int/iris/handle/10665/331656 (accessed on 20 March 2020).
